# Severe SARS-CoV-2 Infection in a Pediatric Patient Requiring Extracorporeal Membrane Oxygenation

**DOI:** 10.1155/2020/8885022

**Published:** 2020-10-09

**Authors:** Shannon M. Flood, Christina M. Osborne, Blake Martin, S. Christopher Derderian, Erin Stenson, Joseph A. Grubenhoff

**Affiliations:** ^1^Children's Hospital Colorado, Section of Emergency Medicine, Aurora, CO, USA; ^2^University of Colorado, School of Medicine Department of Pediatrics, Aurora, CO, USA; ^3^Children's Hospital Colorado, Section of Infectious Diseases, Aurora, CO, USA; ^4^Children's Hospital Colorado, Section of Critical Care Medicine, Aurora, CO, USA; ^5^Children's Hospital Colorado, Department of Pediatric Surgery, Aurora, CO, USA

## Abstract

The overwhelming majority of pediatric cases of SARS-CoV-2 infection are mild or asymptomatic with only a handful of pediatric deaths reported. We present a case of severe COVID-19 infection in a pediatric patient with signs of hyperinflammation and consumptive coagulopathy requiring intubation and extracorporeal membrane oxygenation (ECMO) and eventual death due to ECMO complications.

## 1. Introduction

In late 2019, a novel coronavirus, SARS-CoV-2, was discovered to cause disease in humans with subsequent spread worldwide. Reported adult mortality rates for COVID-19, disease due to infection with SARS-CoV-2, range from 2.0 to 4.4%, and case fatality rate increases with increasing age and comorbidities [1]. To this point, pediatric infection with SARS-CoV-2 appears to be more mild in comparison with fewer reports of critically ill children and few reports of children requiring extracorporeal membrane oxygenation (ECMO) [2–4]. We present a case of a 16-year-old female with multiple chronic medical problems who developed acute respiratory distress syndrome (ARDS) with SARS-CoV-2, was cannulated to extracorporeal membrane oxygenation (ECMO), and ultimately died due to complications related to ECMO.

## 2. Patient Information

The patient was a 16-year-old Latinx female who presented to the Emergency Department (ED) with chief complaints of dyspnea, cyanosis, and fever in the setting of five days of cough, congestion, and lethargy. Her past medical history was notable for remote history of left hemispheric glioma at 18 months treated with chemotherapy, radiation, and debulking, in remission since 2012 with subsequent epilepsy, global developmental delay, and obstructive sleep apnea without baseline need for respiratory support. On presentation, she was afebrile, tachypneic with oxygen saturation (SpO_2_) of 70% on room air, and minimally responsive to sternal rub. She appeared pale with delayed capillary refill, and breath sounds were coarse bilaterally with diminished breath sounds accompanied by severe retractions. Vital signs were notable for respiratory rate 29, heart rate 118, and pressure at 103/65 mmHg. The remainder of exam was unchanged from her baseline.

## 3. Emergency Department Course

**Figure 1:**
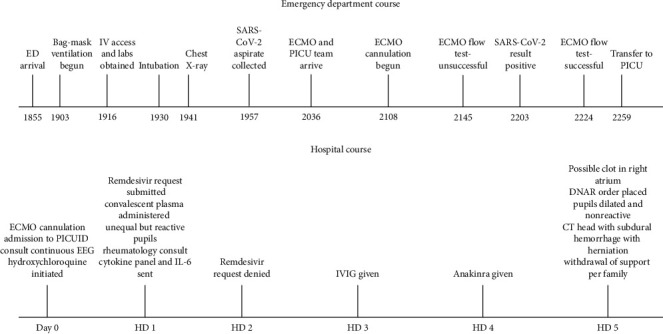
Emergency department timeline of significant events upon initial presentation and hospital course by day in the PICU.

**Figure 2:**
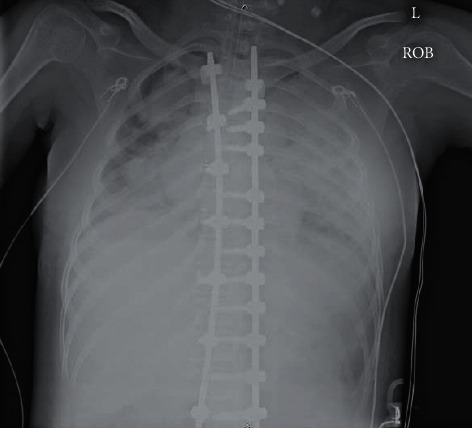
Initial chest X-ray demonstrated near-complete airspace consolidation on the left and patchy consolidations on the right.

On presentation, resuscitation efforts followed standard advanced life support guidelines (Figure 1) including intravenous hydration and administration of antibiotics for suspected sepsis. Bag-mask ventilation was initiated with poor lung compliance and ongoing hypoxemia despite high inspiratory pressures. The patient was subsequently intubated successfully, but SpO_2_ remained between 70 and 85%. Initial venous blood gas was notable for respiratory acidosis (pH of 7.16; pCO_2_ of 67 mmHg). Chest radiograph demonstrated near-complete airspace consolidation on the left and patchy consolidations on the right (Figure 2). Her SpO_2_ remained between 70 and 85% despite administration of 100% FiO_2_ and positive end expiratory pressure titrated up to 14 cmH_2_O. Suctioning produced a small amount of bloody secretions followed by a precipitous decline in SpO_2_ to 50–60%. Due to inability to oxygenate appropriately as well as the need for significant vasoactive inotropic support, the decision was made to cannulate to venoarterial (VA) ECMO prior to transfer to the pediatric intensive care unit (PICU). The patient was cannulated to ECMO by a General Surgery in the ED, and initial inadequate circuit flows due to ECMO cannula positioning in the internal jugular vein were improved with subsequent placement in the right femoral vein.

Initial laboratory studies were notable for hyponatremia, hypochloremia, hypoalbuminemia, signs of coagulopathy, elevated lactate dehydrogenase, normal troponin, thrombocytopenia, leukocytosis with a left shift, and signs of profound systemic inflammation (Table 1). Nasopharyngeal aspirate was positive for SARS-CoV-2 (Cepheid Xpert Xpress Sunnyvale, CA). Respiratory pathogen panel (Biofire FilmArray Respiratory Panel 2.0, Salt Lake City, UT) was negative. Blood and urine cultures were negative, and a tracheal aspirate culture grew mixed upper respiratory flora. Further head imaging was held based on the patient's initial nonfocal neurologic exam and in order to prioritize placing the patient on the ECMO circuit and transferring to the ICU.

## 4. Pediatric Intensive Care Unit Course

The patient was sedated and intermittently neuromuscularly relaxed. She was noted to have anisocoria, left pupil greater than right, with reactive pupils. When not neuromuscularly relaxed, she moved all extremities equally with intermittent purposeful movements and a nonfocal neurologic exam. Head imaging was deferred given the patient's otherwise nonfocal neurologic exam, reassuring cerebral near-infrared spectroscopy, NIRS, and risk of travel to CT scanner. Chest radiography throughout admission demonstrated persistent bilateral, multifocal opacities consistent with reports of severe COVID-19 and ARDS. Echocardiogram demonstrated mild-to-moderately diminished left ventricular function.

Throughout her hospital course, she was continued on mechanical circulatory support with VA-ECMO, and blood flow and sweep gas flow rate were titrated to maintain normal tissue oxygen delivery. She was ventilated using a lung-protective strategy with persistent poor lung compliance. Neurologic status was monitored with bilateral cerebral NIRS as well as continuous EEG. She received blood products as needed, and parameters were chosen to align with published guidelines for COVID-positive patients [5].

The patient received a 3-day course of hydroxychloroquine and underwent baseline and daily evaluation of QTc interval as well as laboratory monitoring to evaluate for drug reaction. She did not meet criteria at the time for compassionate use of remdesivir due to requirement of vasopressor support and VA-ECMO. She received matched convalescent SARS-CoV-2 plasma from the hospital's blood bank which was given following local institutional review board approval as an emergency investigational new drug with consent from the patient's mother.

The patient demonstrated ongoing signs of systemic inflammation, presence of hypogammaglobulinemia, and signs of macrophage activation including hyperferritinemia, thrombocytopenia, and elevated LDH. Cytokine studies were notable for significantly elevated levels of inflammatory cytokines (Table 1). The patient received 1 g/kg of intravenous immune globulin (IVIG) and anakinra. Overall, the patient tolerated these medications well without overt adverse effects. Following administration of IVIG, she had improvement in her C-reactive protein (CRP). With respect to antibiotics, she continued to receive ceftriaxone for empiric treatment of possible superimposed bacterial pneumonia throughout her hospital course.

On hospital day 5, the patient's right pupil became fixed and dilated. A noncontrast head CT demonstrated a large left subdural hemorrhage causing significant uncal and subfalcine herniation along with leftward midline shift and compression of the right cerebral hemisphere. Due to the expected poor prognosis, the family elected to withdraw life-sustaining support.

## 5. Discussion

Reports to date on SARS-CoV-2 infections in children suggest that symptoms are most often mild [2]; however, cases of severe illness and death have been published [3, 6]. There have been no published case reports of use of ECMO in children with COVID-19; however, per registry data, two patients have been successfully weaned from ECMO in Europe, and one received ECMO in North America without outcome available at this time [3, 7].

Our patient had severe COVID-19 with respiratory failure and need for emergent intubation and cannulation to ECMO. No data related to prognosis for intubation and need for ECMO exist in the pediatric population related to COVID-19. However, this patient's initial laboratory studies including profoundly elevated CRP, LDH, D-dimer, elevated ferritin, thrombocytopenia, and initial significant left shift with subsequent leukopenia and lymphopenia have all been associated with more severe disease in adult patients with SARS-CoV-2 infection [8]. The patient demonstrated signs of cytokine storm that have been described in patients with severe COVID-19 including hyperinflammation, signs of macrophage activation, and evidence of progressive consumptive coagulopathy. Furthermore, she had evidence of derangements of inflammatory cytokines associated with severe COVID-19 and macrophage activation syndrome including profoundly elevated levels of IL-6, IL-18, and soluble IL-2 receptor [9].

With respect to treatment, our patient was not granted use of remdesivir, an antiviral that is beginning to show potential to shorten the duration of symptoms in patients with severe SARS-CoV-2 infection [10], due to vasopressor support and ECMO being exclusions at the time. She received a course of hydroxychloroquine, but this has subsequently been shown to have no efficacy and carries risks of cardiac sequelae. Convalescent plasma has been initiated as therapy for severe cases of COVID-19 based on evidence of safety and efficacy in small patient populations with severe viral infections including SARS-CoV-1 and influenza [11], with emerging evidence for decreased viral load and mortality in SARS-CoV-2 infections [12]. While not well studied in COVID-19 [13], IVIG has been used in other disease processes marked by profound systemic inflammation, immune dysregulation, and cytokine storm including toxic shock syndrome and Kawasaki disease. Our patient did not receive dexamethasone or other steroid therapies given that at the time of her presentation, the benefits of steroid treatment for cytokine storm associated with COVID-19 were unknown. Most therapies administered for treatment of COVID-19 have little to no evidence, which makes it challenging to determine the safest and most effective treatment plan. Furthermore, access to clinical trials and guidelines for experimental therapies often exclude or do not consider children.

Overall survival for pediatric patients requiring ECMO for respiratory failure has been reported to be 58% [14, 15]. To date, there have been 1,117 adult patients with confirmed SARS-CoV-2 infection managed with ECMO support (median age: 49 years; IQR: 40–57 years) (https://www.elso.org/Registry/FullCOVID19RegistryDashboard.aspx). Of the 479 for whom hospital discharge data were available, 253 (53%) were discharged alive. Adult data suggest that comorbidities including chronic lung disease, heart disease, and obesity increase the risk of severe disease [16].

The hallmark of treatment for ARDS (including due to COVID-19) is vigilant supportive care. In this case, a lung-protective ventilation strategy while on ECMO was utilized while awaiting improvement in respiratory mechanics and native gas exchange. While the prone and lateral decubitus positions appear to be beneficial for oxygenation in adult patients [17], there are not yet data to support this approach in children. Our patient was not placed in the prone position due to her hemodynamic instability and femoral vein cannulation.

Risks of patient- and circuit-related thromboses must be balanced with bleeding risk when considering anticoagulation on ECMO. Unique factors that may have contributed to this patient's intracranial hemorrhage include her history of intracranial malignancy and prior treatment with chemotherapy and radiation, the initial rapid increase in her serum sodium, anticoagulation required for ECMO support, and sacrifice of the right internal jugular vein during the first ECMO cannulation attempt. Intracranial hemorrhage resulting from anticoagulation while on ECMO has been reported to occur in 16% of children placed on ECMO [14]. Descriptions of hemorrhage range from subtle cerebral microbleeds or large subdural hematomas resulting in intracranial herniation. Children appear to be at highest risk the first 4-5 days while on ECMO [18]. A procoagulable state has been reported in adult patients with COVID-19 [19], but further studies will be needed to better delineate the risk of thrombosis in pediatric patients with COVID-19 including in those requiring ECMO support.

The case presented highlights the potential severity of COVID-19 in pediatric patients with complex medical history including the potential need for ECMO and risk of death. Overall, this patient demonstrated multiorgan dysfunction, shock, and cardiac dysfunction related to COVID-19 with evidence of cytokine storm and coagulopathy.

## Figures and Tables

**Table 1:** Hospital course for patient including laboratory studies and medical inventions.

Laboratory studies or intervention	Reference range	Illness day 5	Illness day 6	Illness day 7	Illness day 8	Illness day 9	Illness day 10
		Hospital day 0	Hospital day 1	Hospital day 2	Hospital day 3	Hospital day 4	Hospital day 5
WBC (10^3^/mcL)	5.20–9.70	13.68 ⟶ 5.93	18.74	17.76	14.00 ⟶ 4.98	5.72	4.39
Hbg (g/dL)	11.3–14.7	12.2 ⟶ 13.0	10.4	10.5	10.7 ⟶ 10.1	9.9	10.4
Plt (10^3^/mcL)	150–500	102 ⟶ 70	69	49	96 ⟶ 44	98	104
ANC (10^3^/mcL)	2.00–5.80	4.24 ⟶ 3.62	15.85	16.69	10.64	3.47	2.3
ALC (10^3^/mcL)	1.03–2.18	5.34 ⟶ 0.83	1.11	0.53	1.54	1.07	1.08
Neurophil bands (10^3^/mcL)	0–1.00	3.28 ⟶ 7.95	0	4.62	1.4	0	0
ESR (mm/hr)	0–20		14	32	44	80	55
CRP (mg/dL)	0–0.9	19		24.8	21.2		14.8
PCT (ng/dL)	<0.1	3.1	8.5	7.9	5.8	10.8	
Troponin I (ng/dL)	0–0.119	<0.012		0.049	0.046		
BUN (mg/dL)	8–21	14	3	5	10	19	27
Cr (mg/dL)	0.42–0.90	0.74	0.39	0.37	0.41	0.37	0.41
Albumin (g/dL)	3.7–5.6	2.9	2.6	2.5	2.5	2.5	3.1
AST (U/L)	5–30	77	75	71	89	72	101
LDH (mU/L)	340–670	1564	1514	1607	2205	2019	2020
Lactate (mmol/L)	0.50–2.00	5.6	2.00	1.50	1.10	1.40	1.30
D-dimer (mcg/mL)	≤0.48	3.41	3.76	2.71	1.94	1.55	1.70
PT (seconds)	12.0–15.0	16.3	3.76	17.4	17.8	18.0	16.9
Ferritin (ng/dL)	10–70	309	16.3	438	668	637	637
Fibrinogen (mg/dL)	150–400	500	377	459	486	527	486
Antibodies		Azithromicin, levoflaxin, vancomycin	Ceftriaxone, vancomycin	Ceftriaxone	Ceftriaxone	Ceftriaxone	
Infusions/transfusion		pRBCs	Platelets, 25% albumin	Platelets	pRBCs, platelets	Platelets	
SARS-CoV-2 theraphy			Hydroxychloroquine, convalescent plasma	Hydroxychloroquine	Hydroxychloroquine, IVIG (1 g/kg)	Anakinra	Anakinra
Vasoactive agents		Epinephrin, norepinephrin	Epinephrin	Epinephrin	Epinephrin	Epinephrin	

If multiple iterations of the same laboratory studies were obtained on the same day, the representative results are displayed with “ ⟶ ” in between. WBC: white blood cell count, Hgb: hemoglobin, Plt: platelets, ANC: absolute neurophil count, ALC: absolute lymphocyte count, ESR: erythrocyte sedimentation rate, CRP: C-reactive protein, PCT: procalcitonin, BUN: blood urea nitrogen, Cr: creatinine, AST: aspirate aminotransferase, LDH: lactate dehydrogenase, and PT: prothrombin time.

## Abbreviations

ECMO: Extracorporeal membrane oxygenation
ARDS: Acute respiratory distress syndrome
PICU: Pediatric intensive care unit.

## References

[1] Hu Y., Sun J., Dai Z. (2020). Prevalence and severity of corona virus disease 2019 (COVID-19): a systematic review and meta-analysis. *Journal of Clinical Virology*.

[2] Castagnoli R., Votto M., Licari A. (2020). Severe acute respiratory syndrome coronavirus 2 (SARS-CoV-2) infection in children and adolescents. *JAMA Pediatrics*.

[3] Shekerdemian L. S., Mahmood N. R., Wolfe K. K. (2020). Characteristics and outcomes of children with coronavirus disease 2019 (COVID-19) infection admitted to US and Canadian pediatric intensive care units. *JAMA Pediatrics*.

[4] DeBiasi R. L., Song X., Delaney M. (2020). Severe coronavirus disease-2019 in children and young adults in the Washington, DC, metropolitan region. *The Journal of Pediatrics*.

[5] MacLaren G., Fisher D., Brodie D. (2020). Preparing for the most critically ill patients with COVID-19: the potential role of extracorporeal membrane oxygenation. *JAMA*.

[6] Lu X., Zhang L., Du H. (2020). SARS-CoV-2 infection in children. *New England Journal of Medicine*.

[7] Boast A., Munro A., Goldstein H. (2020). An evidence summary of paediatric COVID-19 literatur. *Don’t Forget the Bubbles*.

[8] Wynants L., Van Calster B., Collins G. S. (2020). Prediction models for diagnosis and prognosis of covid-19: systematic review and critical appraisal. *BMJ*.

[9] Wen W., Su W., Tang H. (2020). Immune cell profiling of COVID-19 patients in the recovery stage by single-cell sequencing. *Cell Discovery*.

[10] Beigel J. H., Tomashek K. M., Dodd L. E. (2020). Remdesivir for the treatment of covid-19—preliminary report. *The New England Journal of Medicine*.

[11] Mair-Jenkins J., Saavedra-Campos M., Baillie J. K. (2015). The effectiveness of convalescent plasma and hyperimmune immunoglobulin for the treatment of severe acute respiratory infections of viral etiology: a systematic review and exploratory meta-analysis. *Journal of Infectious Diseases*.

[12] Qiu H. B., Li X. Y., Du B. (2020). The keypoints in treatment of the critical coronavirus disease 2019 patient(1). *Journal of Tuberculosis and Respiratory Diseases*.

[13] Cao W., Liu X., Bai T. (2020). High-dose intravenous immunoglobulin as a therapeutic option for deteriorating patients with coronavirus disease 2019. *Open Forum Infectious Diseases*.

[14] Zabrocki L. A., Brogan T. V., Statler K. D., Poss W. B., Rollins M. D., Bratton S. L. (2011). Extracorporeal membrane oxygenation for pediatric respiratory failure: survival and predictors of mortality. *Critical Care Medicine*.

[15] Dal Nogare A. R., Toews G. B., Pierce A. K. (1987). Increased salivary elastase precedes gram-negative bacillary colonization in postoperative patients. *American Review of Respiratory Disease*.

[16] Zheng Z., Peng F., Xu B. (2020). Risk factors of critical & mortal COVID-19 cases: a systematic literature review and meta-analysis. *Journal of Infection*.

[17] Alhazzani W., Møller M. H., Arabi Y. M. (2020). Surviving Sepsis Campaign: guidelines on the management of critically ill adults with coronavirus disease 2019 (COVID-19). *Critical Care Medicine*.

[18] Dalton H. J., Reeder R., Garcia-Filion P. (2017). Factors associated with bleeding and thrombosis in children receiving extracorporeal membrane oxygenation. *American Journal of Respiratory and Critical Care Medicine*.

[19] Spiezia L., Boscolo A., Poletto F. (2020). COVID-19-related severe hypercoagulability in patients admitted to intensive care unit for acute respiratory failure. *Thrombosis and Haemostasis*.

